# Thirty-Seven Human Cases of Sparganosis from Ethiopia and South Sudan Caused by *Spirometra* Spp.

**DOI:** 10.4269/ajtmh.15-0236

**Published:** 2015-08-05

**Authors:** Mark L. Eberhard, Elizabeth A. Thiele, Gole E. Yembo, Makoy S. Yibi, Vitaliano A. Cama, Ernesto Ruiz-Tiben

**Affiliations:** Division of Parasitic Diseases and Malaria, Centers for Disease Control and Prevention, Atlanta, Georgia; Ethiopia Dracunculiasis Eradication Program, Federal Ministry of Health, Addis Ababa, Ethiopia; South Sudan Guinea Worm Eradication Program, Ministry of Health, Juba, Republic of South Sudan; The Carter Center, Atlanta, Georgia

## Abstract

Thirty-seven unusual specimens, three from Ethiopia and 34 from South Sudan, were submitted since 2012 for further identification by the Ethiopian Dracunculiasis Eradication Program (EDEP) and the South Sudan Guinea Worm Eradication Program (SSGWEP), respectively. Although the majority of specimens emerged from sores or breaks in the skin, there was concern that they did not represent bona fide cases of *Dracunculus medinensis* and that they needed detailed examination and identification as provided by the World Health Organization Collaborating Center (WHO CC) at Centers for Disease Control and Prevention (CDC). All 37 specimens were identified on microscopic study as larval tapeworms of the spargana type, and DNA sequence analysis of seven confirmed the identification of *Spirometra* sp. Age of cases ranged between 7 and 70 years (mean 25 years); 21 (57%) patients were male and 16 were female. The presence of spargana in open skin lesions is somewhat atypical, but does confirm the fact that populations living in these remote areas are either ingesting infected copepods in unsafe drinking water or, more likely, eating poorly cooked paratenic hosts harboring the parasite.

## Introduction

Sparganosis, or human infection with the larval plerocercoid stage of members of the genus *Spirometra* (Cestoda: Diphyllobothriidea), is a rather common infection in southeast (SE) Asia,^1^–^4^ but is much less frequently reported in other areas of the world, despite being a common infection in cats and dogs throughout much of the world. In Africa, there are limited reports of human cases dating back to 1907 when the first case was noted, and since then there have been sporadic reports and records of human cases although these same authors have speculated that human sparganosis is a relatively common infection, at least in east Africa.^5^–^7^

As the end of the campaign to eradicate Guinea worm disease (GWD) progresses and the number of endemic countries and cases decreases, there is increased effort to detect and contain each case. Concurrent with this is the need to confirm that each case is accurately identified as *Dracunculus medinensis* so that appropriate actions at the country program level can be undertaken. However, not all specimens recovered from or emerging via the skin are *Dracunculus*.^8^,^9^ Up to now, the most common non-*Dracunculus* recovered by national programs and submitted to the World Health Organization Collaborating Center (WHO CC) at Centers for Disease Control and Prevention (CDC) has been *Onchocerca volvulus*. However, over the past 3 years, an increasing number of spargana have been submitted and this report briefly details 37 such cases, two each from 2012 and 2013, and 33 from 2014.

## Case Series Report

**Figure 1.**
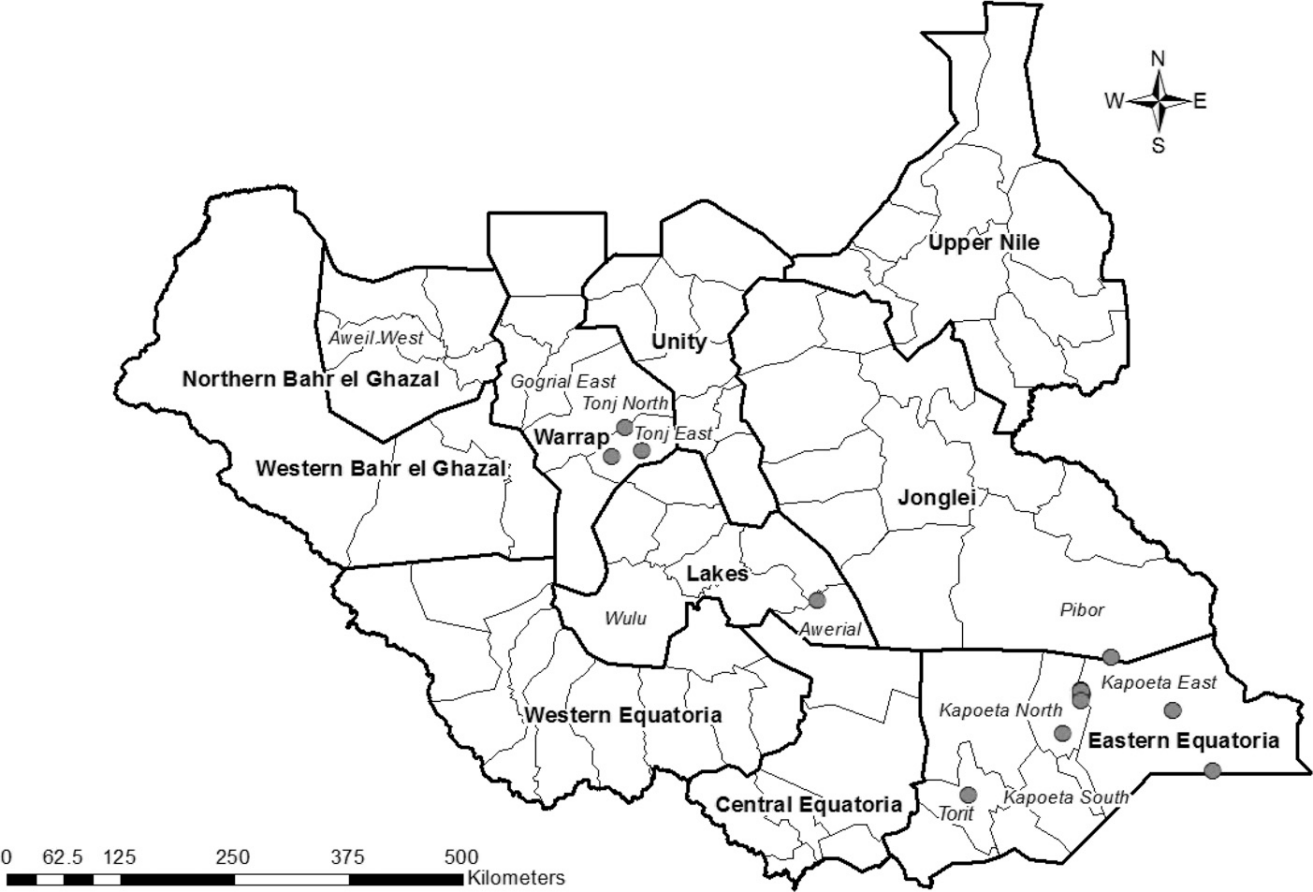
Map of South Sudan indicating the location of 17 villages or village clusters where 34 cases of human sparganosis were detected during 2013–2014.

**Figure 2.**
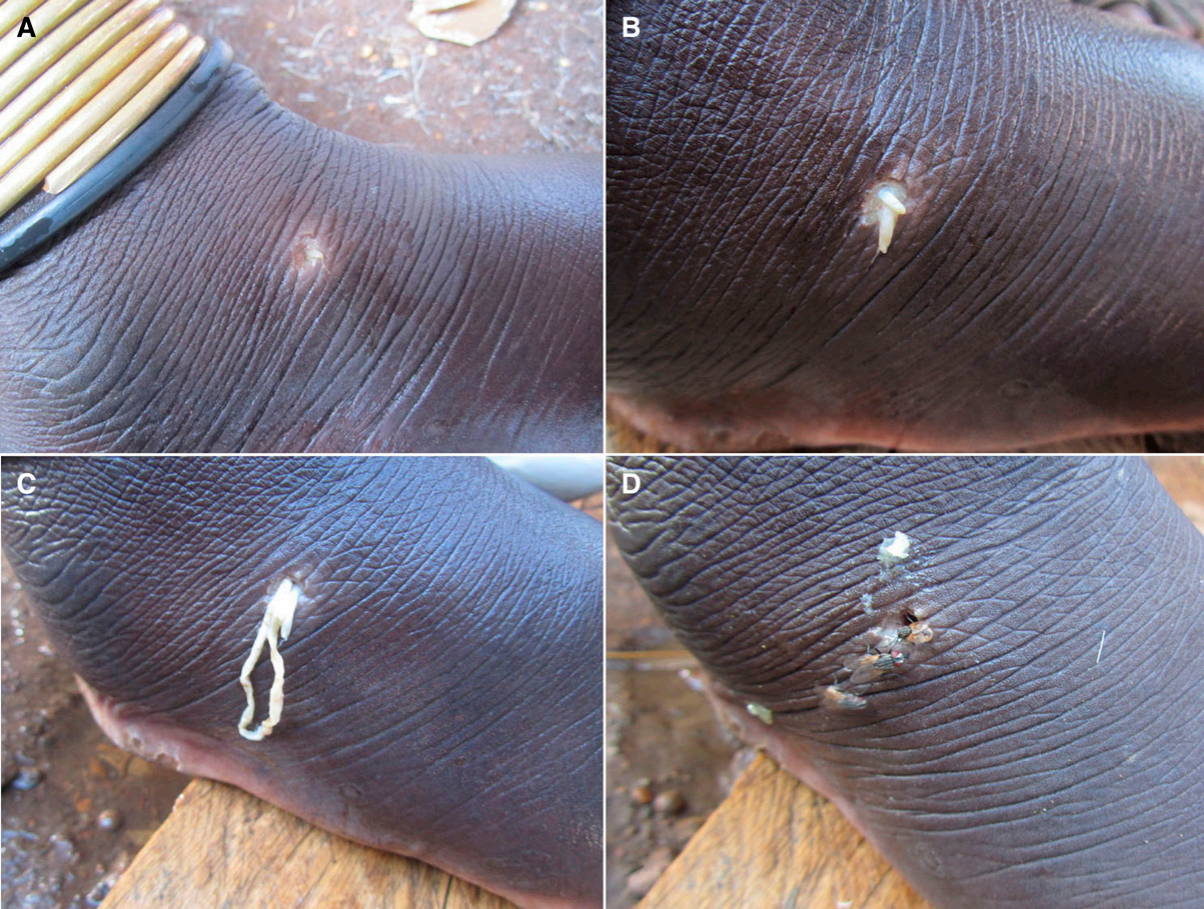
Clinical presentation of a case of sparganosis illustrating the progression of the emergence of the spargana. (**A**) Initial lesion with minute white tip of worm. (**B**) Protrusion of larger portion of larva. (**C**) Mass of “hanging” worm. (**D**) Lesion after final emergence of larva. These images show the remarkable similarity to the appearance of emergence of Guinea worm.

**Figure 3.**
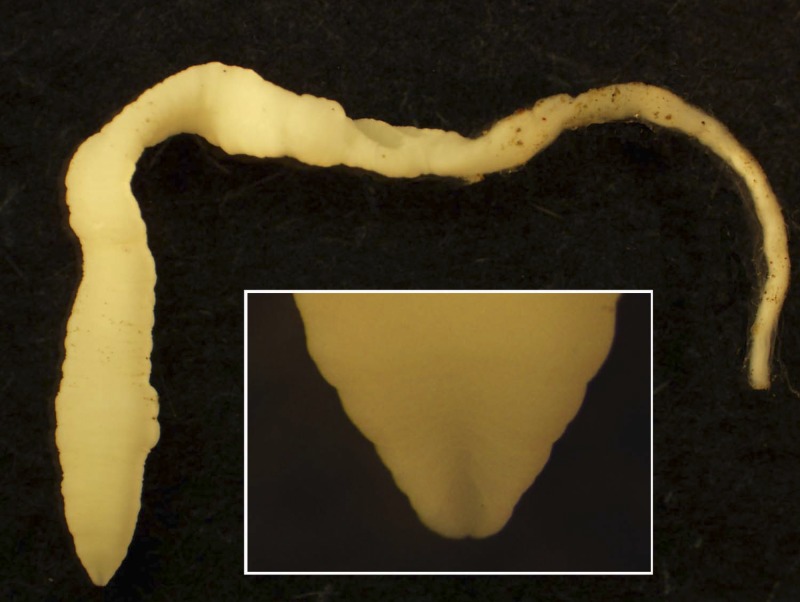
Gross image of entire spargana. Higher magnification showing rudimentary bothrium is shown in inset.

**Figure 4.**
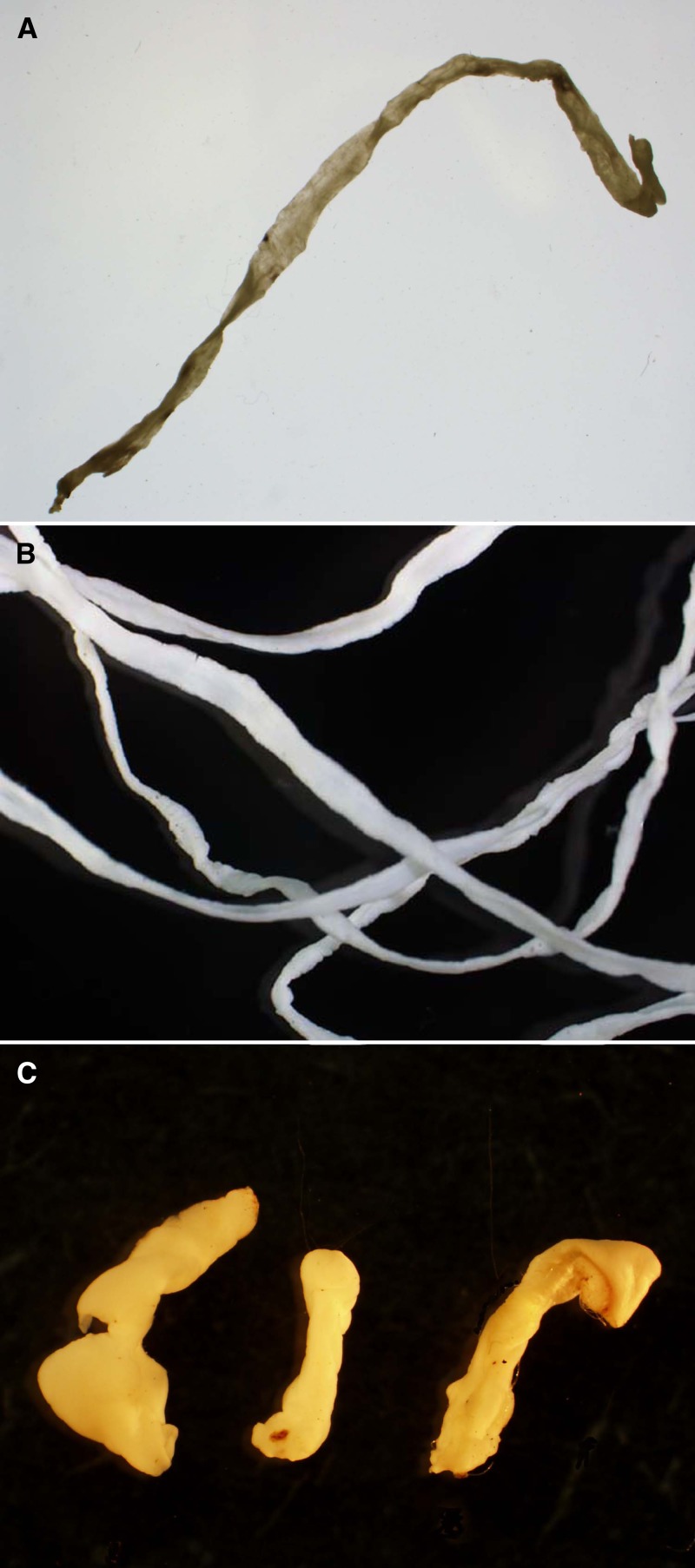
Gross image of three different pieces of spargana that were submitted illustrating the range in size and shape of recovered material.

**Figure 5.**
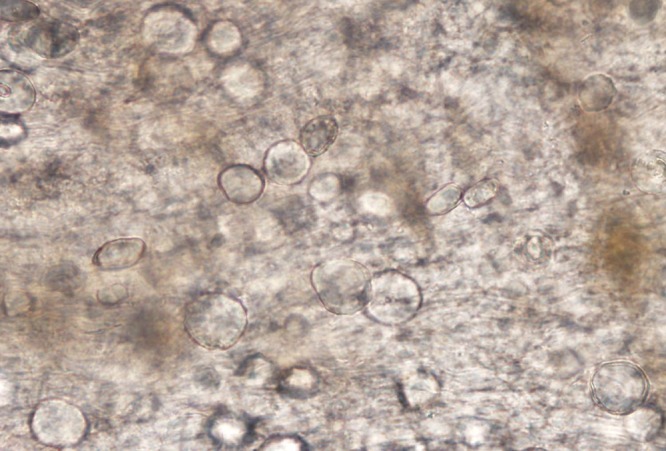
High power image of squash prep of piece of spargana showing the spherical calcareous corpuscles.

As part of routine, ongoing case detection and containment activities in remaining endemic countries, all suspect cases (and rumors) of dracunculiasis are supposed to be investigated within 24 hours. Any emergent worms are collected and preserved in alcohol and sent to CDC for subsequent examination. Thirty-seven of those specimens, three from Ethiopia and 34 from South Sudan, were microscopically identified as spargana; 36 were collected through the national programs to eliminate GWD and one from a surgical procedure for varicose vein. The three specimens submitted from Ethiopia over that time were out of a total of 21 specimens, and for South Sudan, the 34 spargana were out of a total of 161 submitted samples. The cases ranged from 7 to 70 years of age, with a median age of 25 years. Twenty-one (57%) of the cases were male, 16 were female. For the 34 cases from South Sudan, a map showing the location of those cases is provided (Figure 1

). Those 34 cases emerged in 17 different villages, and in those with multiple cases, the number of cases per each individual village were 2, 2, 4, 5, and 9. Specimens were taken from various locations on the body (Figure 2

), including thigh, lower leg, ankle, foot, knee, and penis and measured between 0.5 and 30 cm long, were whitish-tan in color, and often demonstrated a thicker anterior end and a very thin posterior end (Figure 3

). Under low-power microscopic evaluation, rudimentary bothria (cephalic grooves) typical of pseudophyllidian tapeworms was clearly evident on some specimens. However, not all specimens contained the anterior end, and ranged from short to very long ribbon-like pieces of worm (Figure 4

). In those specimens, identification was a bit more challenging, but the presence of calcareous corpuscles (Figure 5

) in the somatic matrix was particularly helpful in establishing that the organism was a larval tapeworm.

Nine of the submitted specimens were subjected to DNA sequencing to further verify the morphological identification. In brief, whole genomic DNA was extracted using a Gentra Puregene Tissue Kit (Qiagen, Frederick, MD) and used for amplification of the nuclear 18S rDNA and mitochondrial cytochrome oxidase 1 (*CO1*) genes. The 18S rDNA amplification was performed with general nematode primers commonly used in molecular verification of *D. medinensis* to confirm that samples were not morphologically atypical guinea worms.^10^ In all nine cases, 18S rDNA reactions were negative, supporting the original diagnosis that specimens were not *D. medinensis*. *CO1* sequences were generated with a custom set of primers designed to serve as a general primer set for diphyllobothriid CO1 amplification. The forward primer sCO1 F (5′-GATAGHMRGGGTGTTGATYTT-3′) and reverse primer sCO1 R (5′-CCARATAGCATGATGCAAA-3′; modified from the diphyllobothriid Cox1Reverse primer of Wicht and others^11^) were used for polymerase chain reaction (PCR) amplification. For cycle sequencing of PCR product, two internal primers (sCO1 intF: 5′-TGTTTACDGTRGGDTTRGAYG-3′ and sCO1 intR: 5′-CAAGCRTAMCCBGACTCRT-3′) were used in addition to the PCR primers so as to ensure complete bidirectional coverage.

Seven of the nine specimens were successfully amplified in 50 μL reactions comprising 1 μL of whole genomic DNA, 1X Platinum^®^ Blue PCR Supermix (Invitrogen, New York, NY), and 0.4 μM of each CO1 primer. Cleaned PCR product (StrataPrep PCR Purification Kit [Agilent Technologies, Foster City, CA]) was sequenced using the BigDye^®^ Terminator v3.1 cycle sequencing kit (Applied Biosystems, New York, NY) and analyzed on a 3130*xl* Genetic Analyzer (Applied Biosystems). Electropherograms were visually inspected and the sequences trimmed and assembled with ChromasPro v1.7.4 (Technelysium, South Brisbane, Australia). High-quality partial *CO1* gene sequences of 996–1,027 base pairs (bp) were generated for the seven successfully amplified specimens and were submitted to GenBank (accession numbers KM248530-248536). Overall, the seven specimens had 99% identity over the 983 bp of shared *CO1* sequence and were polymorphic at 10 single nucleotide sites (average pairwise polymorphism: 2.86 nucleotides, range: 0–8 nucleotides). BLAST queries of the National Center for Biotechnology Information (NCBI) nucleotide database indicated that, of available sequences, the African spargana described here had highest similarity to *Spirometra erinaceieuropaei* (89–90% identity over 983 bp). Significant homologies were also returned for the diphyllobothriid genera *Sparganum*, *Diphyllobothrium*, and *Diplogonoporus* and the cyclophyllid genus *Echinococcus*, with ≥ 91% coverage of African spargana sequences.

**Figure 6.**
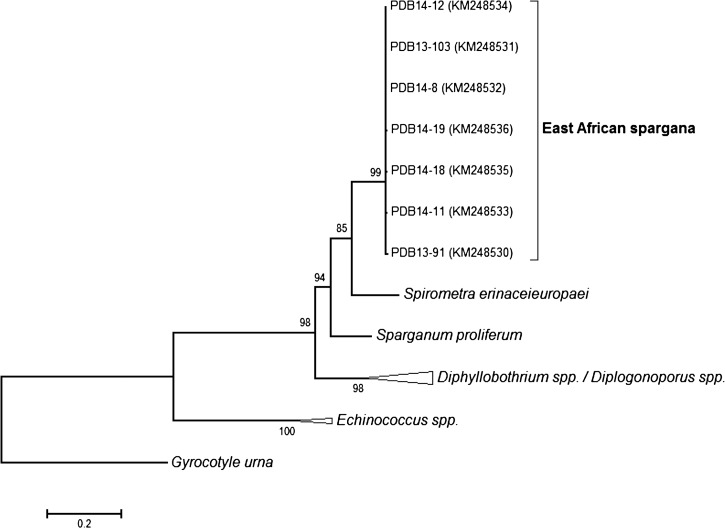
Phylogenetic relationship of seven east African spargana to diphyllobothriid and cyclophyllidean cestodes known or previously reported to infect humans. The tree was inferred with maximum likelihood analysis of partial *CO1* gene sequences (860 base pairs [bp]), using the cestodarian species *Gyrocotyle urna* as the out-group. To focus on the association of the African spargana with other known agents of sparganosis, branches for the *Diphyllobothrium*, *Diplogonoporus*, and *Echinococcus* genera have been collapsed. Branch lengths are shown in number of substitutions per site as indicated by the scale bar, and nodal support is indicated where > 70 (bootstrap, *N* = 1,000). National Center for Biotechnology Information (NCBI) accession numbers of sequences generated in this study are shown in parentheses.

Genetic distances (Kimura 2-parameter model, bootstrap *N* = 1,000) and phylogenetic trees (maximum-likelihood using a GTR+G+I substitution model) were calculated and inferred, respectively, in MEGA v6.0^12^ (freely available at http://www.megasoftware.net) to further investigate the taxonomic relationship among the African spargana, *Spirometra erinaceieuropaei*, and other diphyllobothriid and *Echinococcus* species known or previously reported to occur in humans. Representative *CO1* gene sequences of *Spirometra erinaceieuropaei* (AB015754), *Sparganum proliferum* (AB015753), *Diphyllobothrium ditremum* (FM209182), *D. nihonkaiense* (AB268585), *D. latum* (AB269325), *D. dendriticum* (KC812048), *Diplogonoporus balaenopterae* (AB425839), *Dg. grandis* (AB425840), *Echinococcus canadensis* (AB745463), *E. multilocularis* (AB510023), and *E. granulosus* (GQ168812) were obtained from GenBank, with the cestodarian species *Gyrocotyle urna* (JQ268546) serving as the out-group. Homology of all alignments was supported with amino acid translation before analysis. Average genetic divergence within the African spargana was quite low (*d* = 0.004 ± 0.001) compared with divergence between African spargana and *Spirometra erinaceieuropaei* (*d* = 0.107 ± 0.012) and all other interspecies and intergenus comparisons (*d* ≥ 0.075). Similarly, the inferred phylogenetic tree grouped the seven African spargana into a distinct clade, sister to *Spirometra erinaceieuropaei* within the larger diphyllobothriid clade (Figure 6

).

## Discussion

The life cycle of the Diphyllobothriidea, including *Spirometra*, differs considerably from that of the Cyclophyllidea and typically includes a first and second intermediate host. On reaching water, the egg hatches and releases a ciliated coracidium that is ingested by a copepod, in which the procercoid stage develops. When the infected copepod is ingested by an appropriate second intermediate host, the parasite develops to the plerocercoid stage (= spargana). Birds, reptiles, and amphibians serve as usual second intermediate hosts but many other animals can become infected. If these hosts are eaten by animals other than the normal definitive host, the parasite can infect that animal, but undergoes no further development. Some hosts may serve either as a second intermediate or a paratenic host. When one of these hosts carrying the spargana is eaten by the normal definitive host, a canine or feline depending on the parasite species, the parasite develops into the adult tapeworm in the small intestine. This entire cycle from egg laying to infection in a new definitive host can occur in approximately 3 months, about a quarter of the time required for completion of the Guinea worm life cycle. In contrast to *Diphyllobothrium*, there are some questions about whether fish play a role in the *Spirometra* life cycle and can serve as second intermediate hosts. Humans serve as second intermediate or paratenic hosts, becoming infected with the plerocercoid (spargana) by ingestion of poorly cooked intermediate or paratenic hosts, by using the flesh of an infected second intermediate or paratenic host as poultice on an open wound or the eye, or by ingestion of infected copepods.^1^,^2^ The relative role that each contributes to the overall infection rate is not known.

Despite the dearth of reports of sparganosis in humans from Africa, the occurrence of adult *Spirometra* seems to be both common and widespread, at least in east Africa. Domestic dogs and cats are typical hosts, although Mueller^13^ felt that in Africa, dogs were the preferred host. In addition to domestic animals, wild carnivores are frequent hosts. Hyenas in Kenya and Zambia were noted to have a high rate of infection, with 52/70 (74%) and 2/9 (22%), respectively, of animals examined positive for eggs in the feces,^14^,^15^ and 63% of 112 lions and 87% of 15 lions examined in Tanzania and Zambia, respectively, found to be infected as determined by finding eggs in the feces.^15^–^17^ *Spirometra* was the most commonly found parasite in the examined lions and second most common parasite in the hyenas. The parasite in the hyenas is believed to be *Spirometra pretoriensis* and that from the lions *Spirometra theileri*, although some adult worms from lions could not be distinguished from *Spirometra pretoriensis*, suggesting that both species may occur in lions. Wild herbivores such as antelope, buffalo, zebra, and warthog have been found to be commonly infected with the pleroceroid stage,^5^,^16^ and when these animals fall prey to carnivores such as hyenas and lions, the life cycle is completed.^18^ Opuni and Muller^19^ were able to experimentally infect dogs but not cats with spargana recovered from warthogs in Tanzania. They were also unable to infect various amphibians or reptiles with oral dosing of infected copepods, which calls into question what role typical second intermediate and paratenic hosts such as amphibians and reptiles may play in the life cycle of certain of these African species. Further, it should be noted that at least in east Africa not all recovered tapeworm larvae of the spargana type are actually *Spirometra*. Nelson and others^5^ found that a number of larval tapeworms recovered from various animals were actually tetrathyridia, the larval stage of *Mesocestoides*, and not spargana. Because they resemble each other superficially, some reports of spargana in animals likely represent tetrathyridia. However, larval *Mesocestoides* are not thought to infect humans.

Baboons, vervets, and other monkeys are also infected in east Africa.^20^,^21^ The species infecting monkeys has not been established but is thought to be either *Spirometra theileri*, *Spirometra mansonoides*, or *Spirometra proliferum*, although it could well represent some other species. The range of species of *Spirometra* that occur in Africa is unclear, and given the wide host susceptibility observed in the second intermediate and paratenic hosts, additional work is clearly needed to sort out not only the taxonomy, but also the differences in the life cycle between those species that have different definitive hosts.

When cases of sparganosis are found, microscopy can be the first line of identification. However, further identification beyond that generic determination requires molecular analysis as was the case in this study. The literature to date indicates that the *CO1* gene provides optimal genetic variation for phylogenetic distinction and species-level identification of the Diphyllobthriidea.^22^–^25^ As such, *CO1* sequencing was our chosen method for molecular identification of the unknown spargana recovered in west Africa. *CO1* sequences of the Ethiopian and South Sudanese spargana described here exhibited highest similarity to those of *Spirometra erinaceieuropaei* largely originating from SE Asia. However, both the estimates of sequence divergence and inferred phylogeny suggest that the African spargana reported here are a single sister species to *Spirometra erinaceieuropaei* (Figure 5), though no further designation than *Spirometra* sp. can be applied at this time. Whether the human infections documented in this report represent or are related to species of *Spirometra* known to commonly infect large carnivores in east Africa (e.g., *Spirometra theileri* or *Spirometra pretoriensis*) is unclear and will likely not be fully resolved until further molecular work is done on those species.

It is noteworthy that the life cycle of *Spirometra* utilizes copepods as first intermediate host and various paratenic hosts, very much similar to what was proposed for the recent peculiar epidemiology of GWD in Chad.^26^ The finding of a number of human cases of sparganosis substantiates the potential for such an unusual life cycle for guinea worm to be happening, and suggests that some people in rural and remote areas, at least in South Sudan and Ethiopia, are drinking unprotected water, and, more likely, eating poorly cooked animals that serve as paratenic hosts, such as frogs, snakes, or other animals. Finally, the use of poultices and other natural remedies remains common in remote populations and cannot be discounted as a potential means of infection for some of these cases.
